# The Bacterial Life Cycle in Textiles is Governed by Fiber Hydrophobicity

**DOI:** 10.1128/Spectrum.01185-21

**Published:** 2021-10-13

**Authors:** Andreas Møllebjerg, Lorena Gonzales Palmén, Klaus Gori, Rikke Louise Meyer

**Affiliations:** a Interdisciplinary Nanoscience Center, Aarhus University, Aarhus, Denmark; b Department of Bioscience, Aarhus University, Aarhus, Denmark; c WATEC Aarhus University Centre for Water Technology, Aarhus University, Aarhus, Denmark; d Novozymes A/S, Bagsvaerd, Denmark; University of Minnesota

**Keywords:** biofilms, hydrophobicity, microbiology, skin, textile

## Abstract

Colonization of textiles and subsequent metabolic degradation of sweat and sebum components by axillary skin bacteria cause the characteristic sweat malodor and discoloring of dirty clothes. Once inside the textile, the bacteria can form biofilms that are hard to remove by conventional washing. When the biofilm persists after washing, the textiles retain the sweat odor. To design biofilm removal and prevention strategies, the bacterial behavior needs to be understood in depth. Here, we aim to study the bacterial behavior in each of the four stages of the bacterial life cycle in textiles: adhesion, growth, drying, and washing. To accomplish this, we designed a novel *in vitro* model to mimic physiological sweating in cotton and polyester textiles, in which many of the parameters that influence bacterial behavior could be controlled. Due to the higher hydrophobicity, polyester adhered more bacteria and absorbed more sebum, the bacteria’s primary nutrient source. Bacteria were therefore also more active in polyester textiles. However, polyester did not bind water as well as cotton. The increased water content of cotton allowed some species to retain a higher activity after the textile had dried. However, none of the textiles retained enough water upon drying to prevent the bacteria from adhering irreversibly to the textile fibers. This work demonstrates that bacterial colonization of textiles depends partially on the hydrophobic and hygroscopic properties of the textile material, indicating that it might be possible to direct bacterial behavior in a more favorable direction by modifying these surface properties.

**IMPORTANCE** During sweating, bacteria from the skin enter the worn textile along with the sweat. Once inside the clothes, the bacteria produce sweat malodor and form colonies that are extremely hard to remove by washing. Over time, this leads to a decreasing textile quality and consumer comfort. To design prevention and removal mechanisms, we investigated the behavior of bacteria during the four stages of their life cycle in textiles: adhesion, growth, drying, and washing. The bacterial behavior in textiles during all four stages is found to be affected by the textile’s ability to bind water and fat. The study indicates that sweat malodor and bacterial accumulation in textiles over time can be reduced by making the textiles more repellant to water and fat.

## INTRODUCTION

The characteristic sweat malodor and discoloring of sweaty and used clothes are caused by the colonization and growth of axillary skin bacteria that are introduced during sweating (1). The colonizing bacteria form biofilms that are difficult to remove by conventional washing. As washing is inefficient, the bacteria remain, as do the sweat odor and color. Upon repeated use, the biofilms accumulate, which eventually leads to a decline in textile quality. In order to increase the lifetime and consumer comfort of the textiles, new prevention and removal strategies against textile biofilms are needed. To design biofilm removal and prevention strategies, the bacterial behavior in textiles needs to be understood in depth. The bacterial behavior is expected to be governed by the physicochemical nature of the textile fibers and the textile’s resulting interaction with sweat components. To understand how fiber properties influence bacterial behavior, we studied two textile types with different physicochemical properties. The two most common textile types are polyester and cotton, which may stimulate growth of very different biofilms. Understanding how the biofilms in cotton and polyester differ and which textile properties induce these differences is fundamental for designing new strategies to combat textile biofilms.

**FIG 1:**
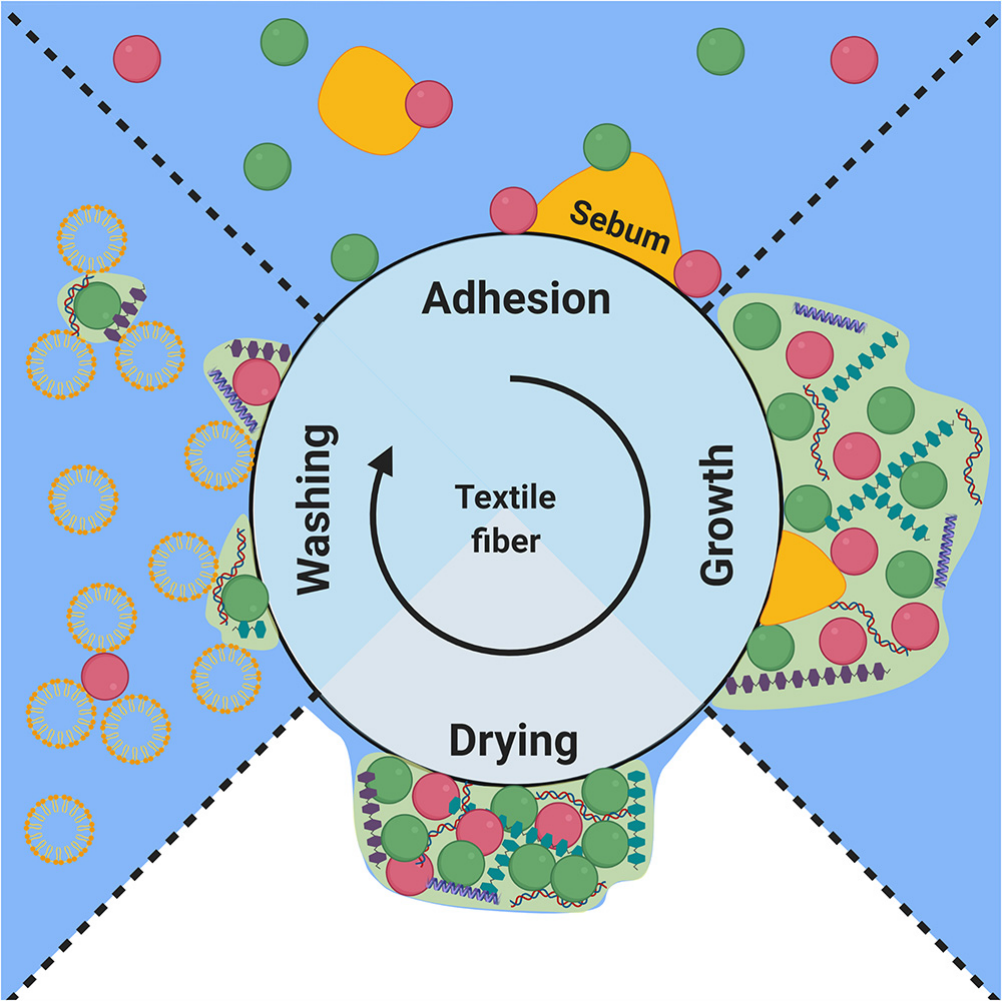
The life cycle of a bacterial biofilm on a textile fiber. The bacteria continuously undergo alternating and periodic stages of adhesion, growth, drying, and washing.

Bacterial colonization and growth in textiles are dynamic phenomena that undergo periodic stages of development as the textile is worn, dried, washed, and worn again (Fig. 1). In the first stage, the axillary bacteria are introduced into the textile during heavy sweating, as occurs during exercise. The sweat is wicked into the textile and carries the suspended bacteria along with it. The bacteria can then adhere to the textile fibers. Bacterial adhesion to a surface is the first step in forming a biofilm, and differences in adhesion to cotton and polyester fibers are likely to influence the resulting biofilm. We hypothesize that biofilms will establish faster on the more adhesive fibers, which will affect the bacterial behavior during the other stages of the life cycle. The physics of bacterial adhesion to surfaces can be described by the extended Derjaguin, Landau, Verwey, and Overbeek (XDLVO) theory (2), which predicts that bacteria will adhere more strongly to more hydrophobic fibers. After adhesion, biofilm growth is limited by the availability of nutrients. The primary nutrient source of bacteria in textiles is the apocrine sweat that is introduced during sweating (1). The composition of apocrine sweat is poorly understood but can be approximated to be a mix of the aqueous eccrine sweat and the lipid sebum (3–5), which have known compositions. Eccrine sweat is composed of ∼99% water, while the residual percentage consists of electrolytes, amino acids, carbohydrates, and vitamins (6, 7). Sebum is composed of triglycerides, fatty acids, squalene, cholesterol, wax esters, and cholesterol esters (7). In this mixture, sebum is thought to be the bacteria’s primary nutrient source. Due to the lipid nature of sebum, it is immiscible in water. As sebum forms a discrete phase in the aqueous sweat, it will not distribute uniformly. The volume and morphology of the absorbed sebum phase depend on the textile properties and will, in turn, affect the growth of the bacteria that metabolize it. The textile type that absorbs more sebum is expected to contain more active bacteria. When sweating ceases, the transport of sweat into the textile ceases as well. As water evaporates from the absorbed sweat, the textile dries and bacterial activity declines. Biofilms that retain water are expected to survive longer than biofilms that dry out. Likewise, drying exposes the bacteria to capillary forces, which can irreversibly adhere them to the textile fibers (8). Textiles that retain water at equilibrium may prevent complete drying and therefore avoid irreversible adhesion during drying. Irreversibly adhered bacteria are very difficult to remove by washing, which poses a big challenge to the laundry industry. The dried biofilm may not be active or viable but can retain malodorous compounds and cause textile discoloring due to bacterial pigments (9). Furthermore, desiccated biofilm can serve as an additional nutrient source of the new axillary bacteria that are introduced once the textile is worn again, which may lead to increased growth and malodor production. Repeated use of the textile will result in accumulating bacterial biomass, decreasing the textile quality and consumer comfort. To design prevention and removal techniques, we need to understand the phenomena that dictate behavior in each stage of the bacterial life cycle (Fig. 1). It is important to note that the stages do not happen in sharply defined time periods for all bacteria and some of the processes in the different stages occur simultaneously. However, dividing the bacterial life cycle in textiles into four stages is a useful simplification when investigating bacterial behavior. Here, we aim to unveil how the bacterial behavior is governed by textile properties.

To investigate the colonization of textiles by axillary bacteria, we created an *in vitro* model in which most of the relevant variables can be controlled. Artificial sweat was mixed *de novo* from the known components (Table 1), while artificial sebum was obtained by a lipid extraction of abdominal subcutaneous fat (10). The model axillary microbiome was composed of equal concentrations of Staphylococcus epidermidis, Staphylococcus hominis, Micrococcus luteus, Corynebacterium jeikeium, and Cutibacterium acnes. These species are found in high density on the axillary skin and are thought to be the main culprits in sweat malodor and discoloring (11–15). The bacteria were adjusted to 2.22 × 10^8^ ml^−1^, which is the estimated concentration of bacteria on the skin when exercise commences, based on an assumed sweating rate of 300 ml m^−2^ h^−1^ and a bacterial surface density of 10^7^ cm^−2^ (11, 16). The textiles were inoculated with the model microbiome and subsequently incubated at controlled temperature and relative humidity to mimic the gradual drying of the textile after wear and prior to washing. The model was used to investigate bacterial adhesion and metabolic activity during the first 3 stages of the bacterial life cycle and to quantify bacterial retention during washing, the last step of the cycle. Although the model has limitations like any model system, the results generated through its use corresponded well with the findings of other studies.

**TABLE 1:** Composition of artificial sweat-sebum

Component	Amt
Amino acids (g/liter)^*a*^^,^^*b*^	
l-Alanine^*a*^	5.16 × 10^−2^
l-Arginine^*a*^	1.36 × 10^−1^
l-Asparagine^*b*^	3.08 × 10^−2^
l-Aspartate^*b*^	8.25 × 10^−2^
l-Citrulline^*a*^	7.01 × 10^−2^
l-Cysteine^*b*^	3.90 × 10^−3^
l-Glutamate^*a*^	5.44 × 10^−2^
l-Glutamine^*b*^	4.50 × 10^−3^
Glycine^*a*^	2.93 × 10^−2^
l-Histidine^*a*^	8.07 × 10^−2^
l-Isoleucine^*a*^	2.23 × 10^−2^
l-Leucine^*a*^	2.75 × 10^−2^
l-Lysine monohydrochloride^*a*^	2.74 × 10^−2^
l-Methionine^*b*^	8.40 × 10^−3^
l-Ornithine monohydrochloride^*a*^	2.53 × 10^−2^
l-Phenylalanine^*a*^	2.13 × 10^−2^
l-Serine^*b*^	4.24 × 10^−1^
l-Threonine^*a*^	5.36 × 10^−2^
l-Tryptophan^*a*^	1.12 × 10^−2^
l-Tyrosine^*a*^	3.08 × 10^−2^
l-Valine^*a*^	2.93 × 10^−2^
Carbohydrates (g/liter)^*b*^	
d-Glucose	2.77 × 10^−1^
Lactic acid (88%)	1.74 × 10^0^
Sodium pyruvate	6.97 × 10^−2^
Nitrogenous compounds (g/liter)^*b*^	
Creatinine	6.00 × 10^−3^
Urea	1.59 × 10^0^
Vitamins (g/liter)^*a*^	
Ascorbate	1.73 × 10^−3^
Choline chloride	3.63 × 10^−3^
Folic acid	7.06 × 10^−6^
Inositol	2.88 × 10^−4^
Nicotinic acid^*c*^	5.05 × 10^−1^
p-Aminobenzoic acid	9.73 × 10^−6^
Pantothenic acid calcium salt	2.48 × 10^0^
Pyridoxine hydrochloride	2.06 × 10^-6^
Riboflavin^*c*^	4.00 × 10^-2^
Thiamine hydrochloride	1.69 × 10^−1^
Inorganic compounds (g/liter)^*b*^	
Calcium sulfate	1.32 × 10^−1^
Potassium hydrogen carbonate	2.60 × 10^-2^
Sodium chloride	6.31 × 10^0^
Sodium phosphate monobasic	4.08 × 10^-3^
Lipids (ml/liter)^*b*^	
Abdominal subcutaneous fat extract	1.64 × 10^1^
Cholesterol	8.00 × 10^−1^
Squalene	2.00 × 10^0^

a See reference 5.

b See reference 10.

c Different from cited.

## RESULTS

### Polyester is more hydrophobic than cotton.

**FIG 2:**
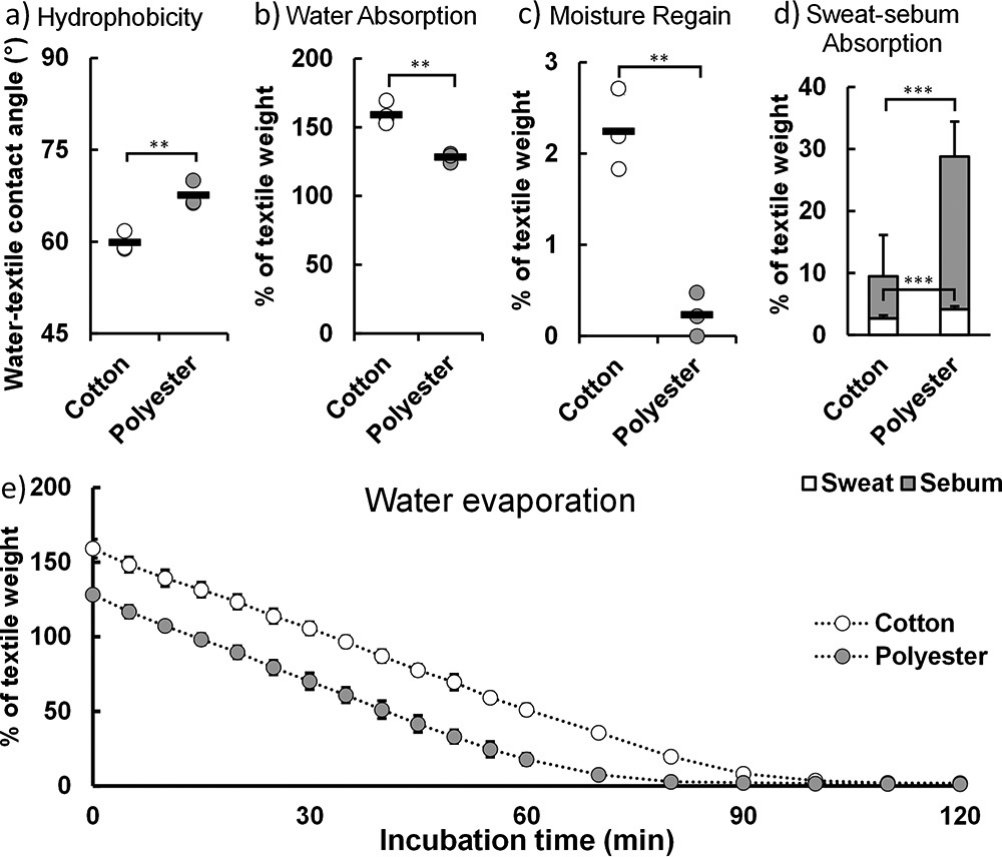
Physicochemical properties of cotton and polyester textiles. (a) Hydrophobicity of cotton and polyester textiles measured by the contact angle with water (*n* = 3). (b) Absorbed water content in cotton and polyester as measured by percentage of textile dry weight (*n* = 4). (c) Moisture regain of cotton and polyester after 24 h (*n* = 3). (d) Absorbed sweat (*n* = 6) and sebum (*n* = 10) in cotton and polyester textiles. (e) Evaporation of inoculated liquid from textile over time (*n* = 4). Statistical significance was evaluated by a two-tailed Welch *t* test.

The physicochemical properties of cotton and polyester are thought to govern the bacterial behavior in all stages of the biofilm life cycle. The hydrophobicity of the textile and the resulting interaction with water and sweat-sebum were investigated. Measuring the contact angle through the Wilhelmy plate method revealed that polyester is significantly more hydrophobic than cotton, differing by ∼8° in the contact angle (Fig. 2a). Previous measurements indicated a difference in contact angle of ∼14° (17). However, neither of the measured contact angles is statistically significantly different from the literature values.

Due to the difference in hydrophobicity, cotton and polyester differ in their hygroscopic behavior. Cotton initially absorbed more water than polyester when inoculated with artificial sweat-sebum (Fig. 2b), indicating that higher hydrophilicity of cotton resulted in a higher hygroscopicity. While cotton and polyester differed in their initial water content, the evaporation kinetics of the textiles were similar (Fig. 2e) and consistent with previous findings (18). Cotton and polyester had initial evaporation rates of 1.78% and 1.85% min^−1^, respectively. The drying process took a little longer in cotton due to the higher initial water content, and cotton also displayed a higher moisture regain at the end of the drying process (Fig. 2c). When inoculated with artificial sweat-sebum, cotton retained 2.24% of the textile weight in water while polyester retained 0.23% water. These results are consistent with earlier findings that show that the moisture regain under these conditions is ∼4% for cotton (19), while it is practically zero in polyester (20). The hygroscopic and hydrophobic properties of the textile can affect bacterial attachment directly and bacterial growth indirectly through its effect on water retention and availability of nutrients from the hydrophobic sebum. We therefore proceeded to investigate the interaction of sebum lipids with the textile fibers.

In contrast to water absorption, the hydrophobic polyester absorbed more sebum and more sweat solutes compared to cotton (Fig. 2d). Liquid chromatography-mass spectrometry (LC-MS) revealed that the sebum was mainly composed of triglycerides (Fig. S1) and contained small amounts of sphingomyelin and phosphatidylcholines originating from the adipocyte cell membranes. Gas chromatography-flame ionization detection (GC-FID) on FAME indicated that the triglycerides primarily contain myristic acid, palmitic acid, palmitoleic acid, stearic acid, oleic acid, and linoleic acid (Fig. S1), indicating that the majority of the sebum components can serve as nutrients to the bacteria. The larger amount of bacterial nutrients will likely stimulate bacterial metabolism in polyester if the distribution of sebum in the textile makes it available to adsorbed bacteria.

### Sebum is distributed along the fiber surface in polyester.

**FIG 3:**
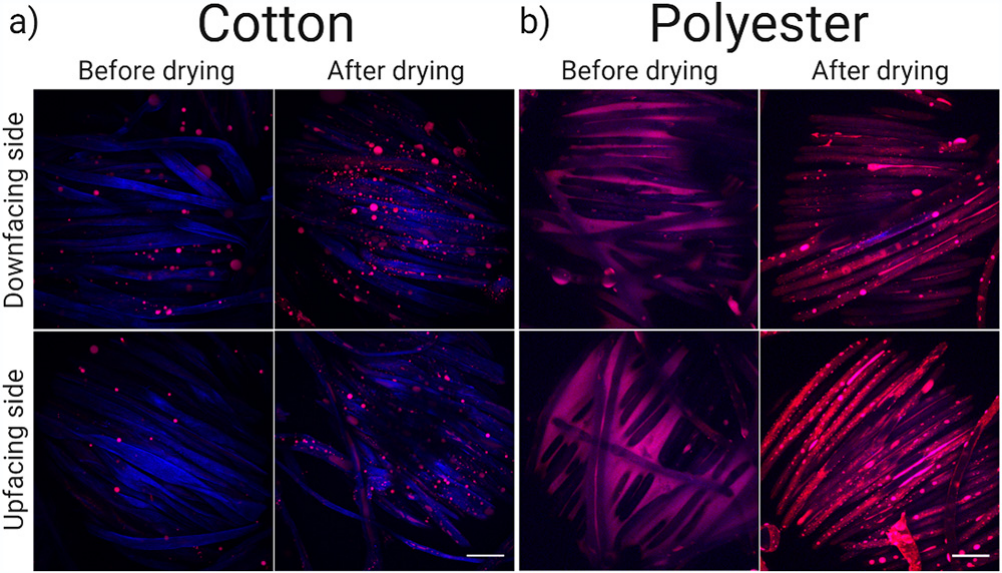
Sebum distribution in cotton (a) and polyester (b) textiles. The top row shows CLSM images of the downfacing side of the textile, before and after drying, while the bottom row shows the upfacing side. The sebum is stained by Nile Red while the fibers autofluoresce in the blue region of the spectrum.

After quantification of sebum absorption, we visualized its distribution to get more insight into sebum’s availability to bacteria in the textile. Specific staining of sebum by Nile Red (Fig. S2) revealed formation of spherical sebum droplets in cotton, and the distribution appeared unaffected by drying the textile (Fig. 3a). In polyester, however, sebum was much more homogenously distributed. It coated the fibers and spanned the space between fibers. Upon drying, sebum remained associated along the length of the fibers. (Fig. 3b). There were no apparent differences between the downfacing and upfacing sides. There were no apparent differences between the sides of the textile that were downfacing or upfacing during contact with the sebum. The images support our finding that the hydrophobic polyester absorbs more sebum than cotton, and they reveal that the absorbed sebum is homogenously distributed in the textile, which potentially increases its availability to bacteria in the textile.

### More bacteria adhere to polyester.

**FIG 4:**
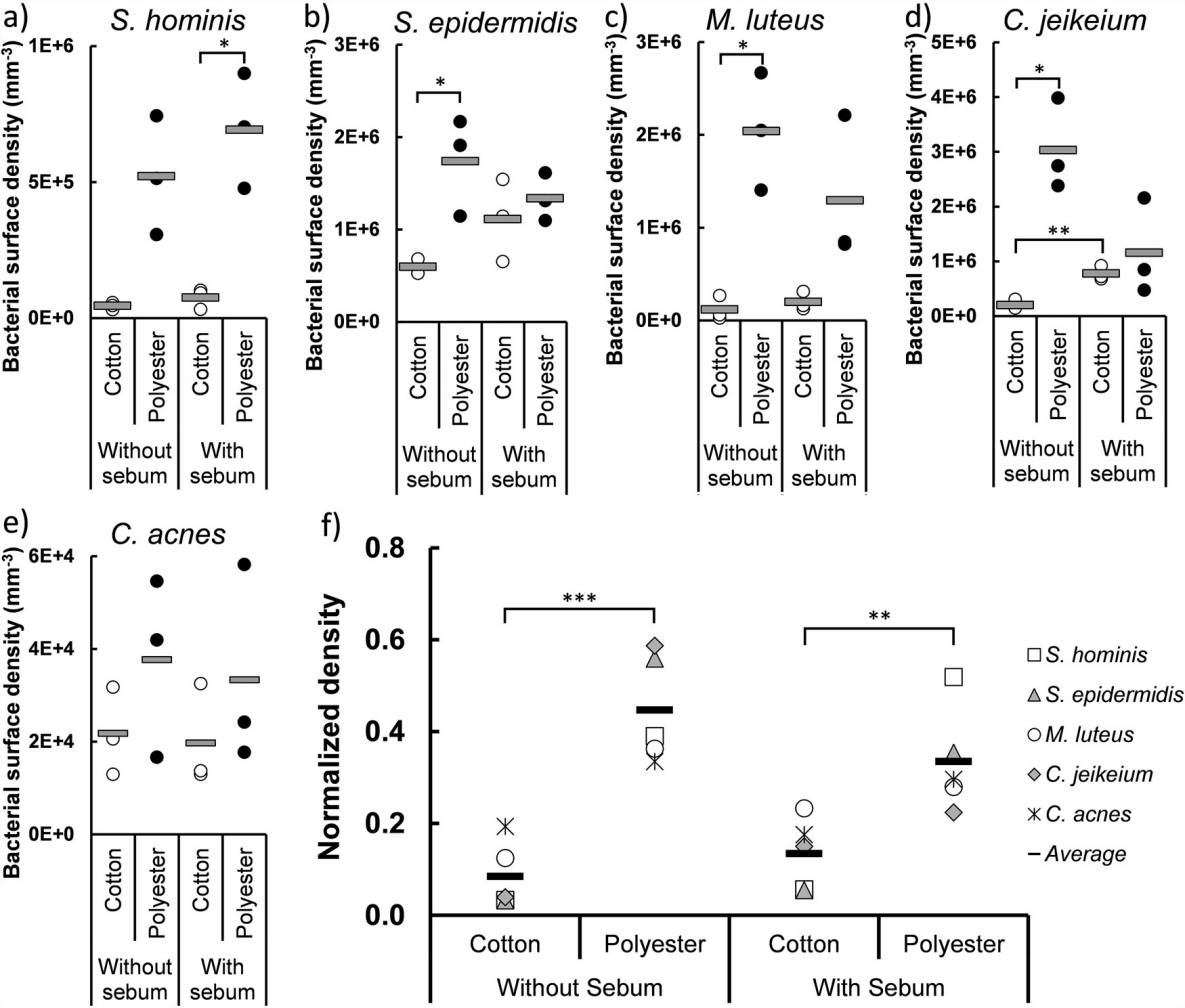
Bacterial adhesion to cotton and polyester textiles preinoculated either with sweat or with sweat and sebum. (a to e) Adhesion of five species to cotton and polyester. Adhesion is indicated by the number of bacteria left per cm^3^ of textile after washing with Triton X-100 (*n* = 3). (f) The surface densities of the single species were normalized and summarized to allow for comparison. Statistical significance was evaluated by a two-tailed Welch *t* test.

The first stage in the life cycle is bacterial adhesion to the textile fibers. The simplest case of adhesion is between bacteria and the naked textile fibers. When the bacteria were incubated with the textile without sebum, all species favored adhesion to polyester (Fig. 4). Only C. acnes, which adhered poorly to both textile types, did not display preferential adhesion to one over the other.

During sweating, the absorbed sebum affects the surface properties of textile fibers and may therefore influence the bacterial adhesion. To quantify sebum’s role in bacterial colonization of textiles, we compared bacterial colonization of cotton and polyester before or after adsorption of sebum in the textile. Collectively, the presence of sebum did not influence bacterial adhesion to either textile type. Only a small effect was seen for C. jeikeium, which adhered better to cotton when sebum was present (Fig. 4d). The textile samples were gently washed after bacterial adhesion, and we speculated that bacteria adhered to adsorbed sebum may be released if sebum desorbed from the textile during the washing step. However, sebum remained in the textile after washing with Triton X-100 (Fig. S3). Hence, bacteria adhere more readily to polyester, independently of sebum adsorption (Fig. 4), and biofilm formation therefore has a head start in polyester textiles.

### Bacteria are initially more active in polyester.

**FIG 5:**
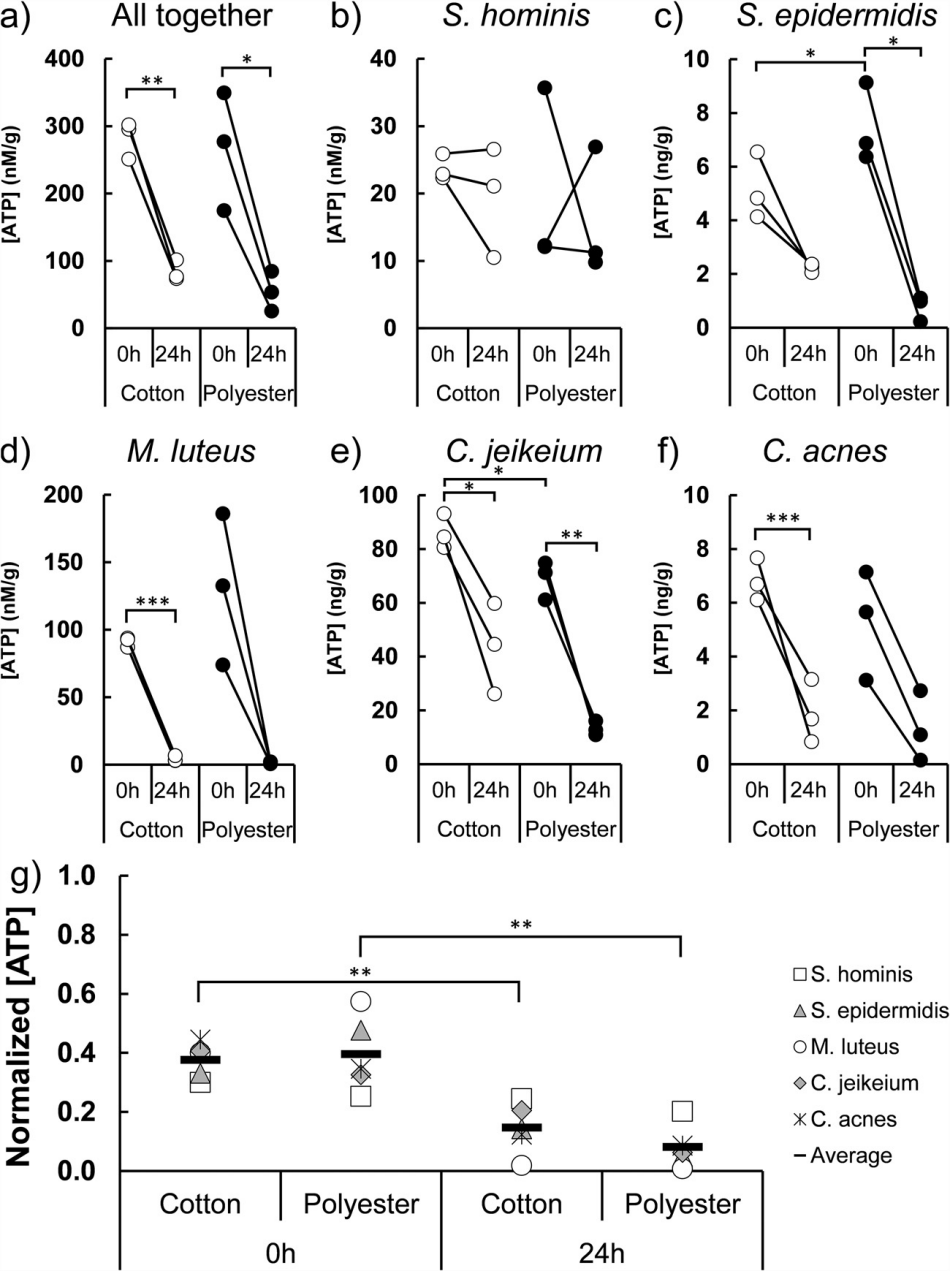
Bacterial activity in textiles. (a to f) ATP concentration was measured before and after 24 h of incubation (*n* = 3). (g) The ATP concentration is normalized and summarized to allow for comparison. Statistical significance was evaluated by a two-tailed Welch *t* test.

Once bacteria are adhered to the textile surface, the next stage of the bacterial life cycle is growth. To assess the overall metabolic activity of the adhered bacteria, we measured the ATP content in the textiles inoculated with artificial sweat-sebum and bacteria both before and after the 24-h incubation step. When all five species were inoculated together, there was no significant difference in activity between bacteria adhered to cotton and those adhered to polyester (Fig. 5a). In order to point out any differences in the metabolic activity of the five bacterial species, we inoculated the textiles separately with each species and quantified ATP before and after the incubation of the textile for 24 h at controlled humidity which resulted in drying. S. hominis is the only species that showed higher initial activity in polyester than in cotton. However, the normalized activity indicates that the bacteria collectively have higher initial activity in polyester than in cotton. The higher ATP levels in polyester might simply reflect the greater number of adhered bacteria.

The difference in ATP was absent when the bacteria were inoculated in the absence of sweat-sebum (Fig. S4), confirming that sebum was used in metabolic processes, which is in accordance with previous studies of the axillary bacterial community (10). Although bacterial activity was initially higher in polyester, this trend disappeared after the textile had dried (Fig. 5g).

After 24 h of incubation, bacterial activity had decreased for most bacteria in both textiles, and comparison of normalized activity for all bacteria showed no significant differences in ATP concentration at the end of the incubation. This trend was not observed in S. hominis and S. epidermidis (Fig. 5b and c). These species maintained or even increased their activity in cotton during the 24-h incubation, despite the loss of 98.6% of the water in the textile (Fig. 2c), indicating a higher tolerance to desiccation. Meanwhile, both C. jeikeium and C. acnes had a higher ATP content in cotton than in polyester after drying.

### Bacteria adhere better to the textile after drying.

**FIG 6:**
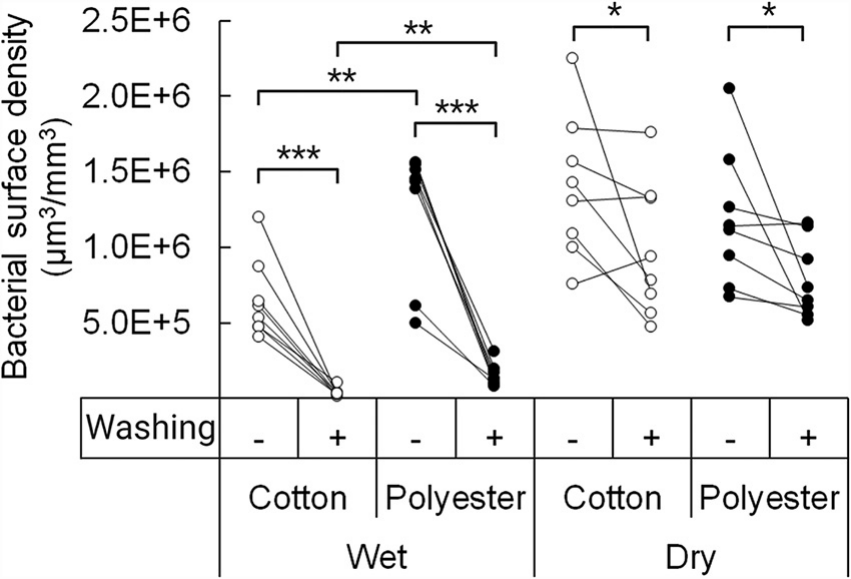
Bacterial adhesion to cotton and polyester before and after drying. The bacterial surface density, measured in μm^3^ bacterial volume per mm^3^ textile, is indicated before and after harsh washing (*n* = 8). Statistical significance was evaluated by a two-tailed Welch *t* test.

In the 24 h between the ATP quantifications, the textiles dried, which influenced the bacterial behavior and adhesion strength. What happens during drying may therefore influence the efficacy of the subsequent washing of the textile. We therefore quantified how many bacteria were retained before and after a harsh washing step carried out on textiles before and after the 24-h incubation. Before drying, both cotton and polyester contained mostly loosely associated bacteria, as indicated by the large decrease in adhered bacteria after washing (Fig. 6). However, polyester maintained a significantly higher quantity of bacteria than cotton, both before and after washing.

The washing efficiency of the textiles decreased significantly after the textiles had dried, as more bacteria became irreversibly adhered as a consequence of drying (Fig. 6). Quantification of bacteria in the dried textiles was highly variable, and we could therefore not show any significant differences between the two textile types.

## DISCUSSION

### Bacteria adhere better to polyester.

The first stage of the bacterial life cycle in textiles is adhesion to the textile fibers. The difference between adhesion to cotton and adhesion to polyester therefore confers an important initial difference on the path to permanent colonization of bacteria in the textile. All the bacterial species tested in this study, except C. acnes, adhered more strongly to the polyester textiles than to cotton (Fig. 4). This corresponds well with the XDLVO theory, which predicts that bacteria adhere more strongly to more hydrophobic surfaces (2). The XDLVO theory is a simplification of bacterial attachment, as it considers bacteria as colloidal particles and predicts their interactions based on average physicochemical properties while ignoring specific interactions of surface appendages that differ from the average cell surface properties. However, the theory does serve as a useful guideline predicting attachment of bacteria to surfaces, especially if the bacteria are nonmotile like the species used in this model (21, 22).

As the bacteria approach the surface, they first enter the secondary energy minimum (23–25), from which they can readily detach from the surface, given sufficient force, such as the shear forces exerted during washing in a laundry machine. Given sufficient energy, the bacteria may also enter the primary energy minimum, in which they become irreversibly attached. Electrostatic interactions play a role in adhesion, but they are often repulsive, as most materials (including cotton and polyester) and most bacteria have negative zeta potentials at neutral pH (25–28). Experiments done under similar ionic strengths indicate that the more influential parameter for bacterial adhesion is hydrophobicity, as acid/base interactions are the dominant forces (25, 29). As polyester is the more hydrophobic textile (Fig. 2a), more bacteria adhere irreversibly to this textile after first entering the clothes during absorption of sweat (Fig. 4 and 6). The same trend was observed previously in other studies when model organisms were used (30, 31). The hydrophilic cotton fibers contain hydrophobic wax impurities that were not completely removed during processing (32). We did not investigate the role of wax residues in this study, but given that hydrophobicity plays a major role in bacterial adhesion to textiles, the amount and distribution of wax residues in cotton are potentially significant for bacterial attachment.

The fiber surface is, however, not the only surface available for colonization in the textile. Absorbed sebum is an additional surface that the bacteria can adhere to. We speculated that the bacteria might adhere primarily to the hydrophobic sebum and that sebum adsorption therefore is a major factor affecting bacterial colonization. However, our study indicates that only *C. jeikeium* adhered better to cotton when sebum was present (Fig. 4d). This is not surprising considering the lipophilic nature of *C. jeikeium* (33). However, we saw no indication that adsorbed sebum promoted bacterial adhesion for the other species in either cotton or polyester (Fig. 4).

### Bacteria attach irreversibly when textiles dry.

**FIG 7:**
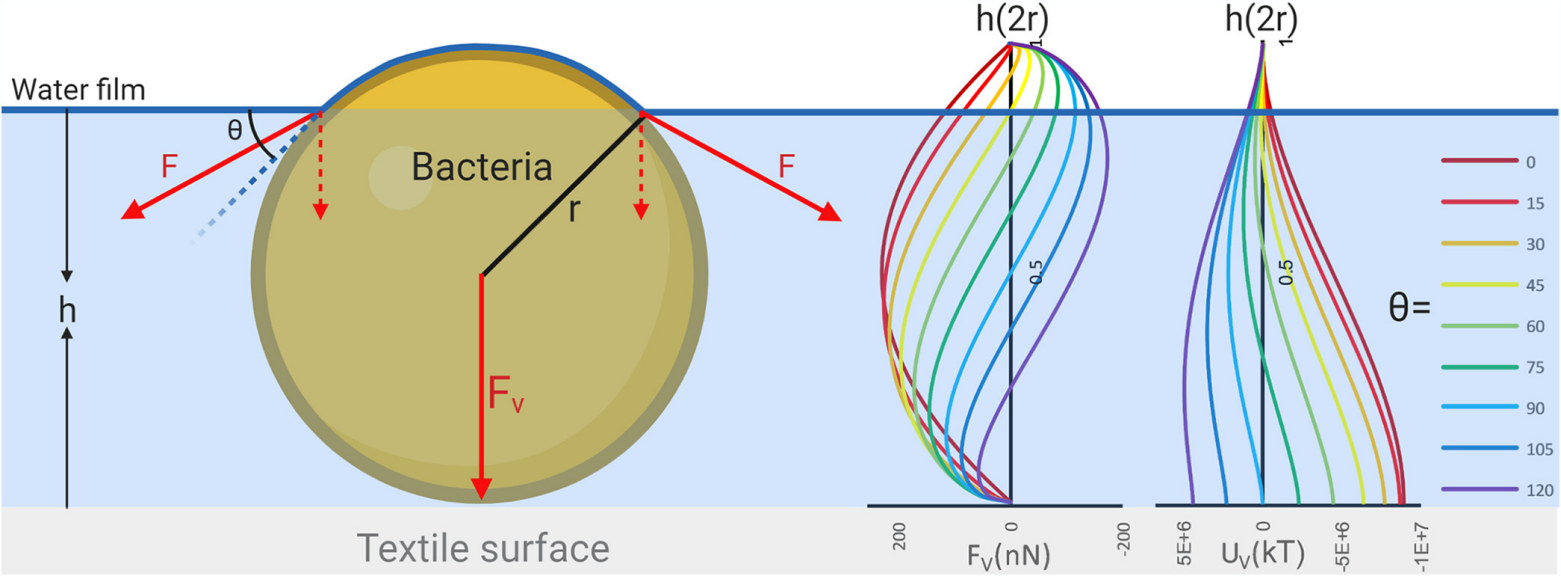
Capillary force acting on a bacterium in a thin water film. The capillary force, *F_v_*, and the resulting potential energy, *U*, are shown as a function of the water film height for a range of water-bacterium contact angles, θ (°). Bacteria with different surface hydrophobicity experience different capillary forces, depending on the water film thickness. Thin water films tend to result in more attractive capillary forces. Adapted from reference 8.

Most bacteria will not adhere irreversibly to the textile fibers while the textile is still wet, because the energy barrier to irreversible adhesion is too high. However, when the textile starts drying, the decreasing water content begins to affect adhesion (Fig. 7). During evaporation, the sweat solutes are concentrated, leading to increasing ionic strength and decreasing electrostatic repulsion between bacteria and textile fiber (25). This effect increases adhesion, independent of textile type. Adhesion during evaporation is dominated by capillary forces (34, 35). As water evaporates, bacteria are caught in a film of water on the surface of textile fibers. When the thickness of the water film is smaller than the diameter of the bacteria, capillary forces immobilize the bacteria on the substrate surface due to formation of a meniscus (8). The capillary force depends on the height of the water film and the water-bacterium contact angle, which reflects the hydrophobicity of the bacterial cell surface (Fig. 7). Hydrophilic bacteria experience the most attractive capillary forces (34). Although hydrophobic bacteria initially experience a repulsive force, the final force upon complete drying is attractive (34). This force is sufficient to drive the bacterium to adhere irreversibly once the water film is thin enough relative to the cell diameter. The capillary force can create a potential that is 2 orders of magnitude larger than the energy barrier to irreversible adhesion created by the XDLVO potential (2). This is likely to make the size of the XDLVO energy barrier (and therefore the hydrophobicity of the surface) irrelevant to adhesion during drying.

The hydrophilic nature of cotton fiber may not influence the adhesion during drying directly, but its effect on hygroscopicity of the textile might. Most of the water in both textiles had evaporated after 2 h (Fig. 2e), but the moisture regain for cotton was 10-fold higher than that for polyester (Fig. 2c). In cotton, the retained water can both be absorbed into the fiber interior (36) and form a hydrating film on the fiber surface. Assuming a fiber diameter of 10 μm, a cotton density of 1.54 g cm^−3^, and that all the water is confined to the surface, the height of the hydrating layer is 170 nm thick after drying under the conditions studied here, meaning that capillary forces are being exerted on the bacteria. Cotton’s hygroscopicity is therefore not sufficient to affect bacterial adhesion in the dry textile under these conditions, and it explains why bacteria remained strongly attached to both cotton and polyester after the textiles were dried (Fig. 6). Inefficient washing with detergent after wear (37, 38) and after drying of textiles incubated with model organisms (39) has previously been observed in cotton and polyester textiles at nonelevated temperatures.

Most textiles are not washed immediately after use, and one would expect that the textile dries before washing. Preventing malodor by modifying textile fibers to decrease bacterial attachment is therefore unlikely to be an effective strategy, as the drying process immobilizes bacteria in the textile regardless of their initial adhesion force. We therefore turn our attention to the properties that affect the bacterial processes causing malodor.

### Polyester provides a readily available nutrient source for microbial growth through sebum adsorption.

The second stage in the bacterial life cycle in textiles is growth. Bacterial growth is influenced by nutrient availability and water content. While cotton absorbed more water (Fig. 2b), polyester absorbed significantly larger volumes of both sebum and sweat solutes (Fig. 2d). The polyester fibers adsorbed more bacteria than cotton, and the initial activity of the adsorbed bacteria was also higher in polyester (Fig. 5g). Teufel et al. also found that an increased amount of bacterial DNA in polyester textiles inoculated and incubated with harvested sweat compared to those in cotton (40). Meanwhile, polyester textiles contained a higher bacterial load than cotton when worn by males for 3 days (37). However, we found that textiles inoculated with bacteria in phosphate-buffered saline (PBS) did not have a higher activity in polyester (Fig. S4). In another study, where textiles were inoculated with bacteria in minimal M9 medium, no increased growth was observed in polyester compared to that in cotton (13). The higher bacterial activity in polyester is observed only when the bacteria are present in the textile with real or artificial sweat-sebum. We therefore ascribe the higher bacterial activity in polyester to the higher sebum content and its accessibility to the adsorbed bacteria.

We use the XDLVO theory again to explain the behavior of sebum lipid particles in the textiles. The hydrophobic sebum had a lower adhesion energy when interacting with polyester than when interacting with cotton, and this drove the spreading of sebum across the polyester surface (Fig. 3b) while confining sebum in cotton to discrete droplets (Fig. 3a). This effect increases the absorption of sebum in polyester (Fig. 2d) and also increases its availability to bacteria by increasing its surface area. Polyester therefore appears to provide optimal access to nutrients for the adsorbed bacteria.

Malodor arises from the activity of bacteria that degrade both eccrine sweat and sebum components in the textile. Degradation of amino acids and lactic acid in sweat produces a range of short-chain (C_2_-C_5_) volatile fatty acids while degradation of fatty acids and triglycerides in sebum result in short-medium-chain (C_2_-C_11_) volatile fatty acids (41, 42). Between eccrine sweat and sebum, sebum is responsible for the majority of the malodor (41, 42). Increased bacterial activity caused by higher sebum availability may therefore explain the higher malodor production previously reported in polyester clothes compared to that in other textiles (3). Decreasing the sebum absorption by increasing hydrophilicity may therefore not only decrease bacterial colonization but also be critical for preventing malodor.

The availability of sebum is not the only parameter relevant to bacterial growth. When sweating ceases and the textile starts to dry out, the textile’s ability to bind water becomes important. Although the evaporation rates in cotton and polyester were almost identical at 37°C and 30% relative humidity (RH) (Fig. 2e), the higher moisture regain in cotton will extend the period that bacteria can grow in the textile. We show that the small amount of water retained in cotton was sufficient for continued activity of the desiccation-resistant staphylococci (Fig. 5b and c).

The higher hygroscopicity of cotton can also affect release of odorous compounds from the textile. Higher moisture regain has previously been correlated to decreased odor production (43). Short-chain fatty acids tend to be very volatile and evaporate from the textile rapidly, while medium-chain fatty acids are less volatile (44). As the water phase evaporates from the textile, the less volatile fatty acids become concentrated, driving further odor release. Cotton can therefore sequester more odor compounds than polyester due to the higher moisture regain (Fig. 2c). In conclusion, the textiles’ interaction with water and sebum will likely lead to a more intense and immediate production of malodor from polyester. The more hydrophilic and hygroscopic textiles like cotton may thus be less prone to malodor production. However, they would also prolong bacterial activity and survival in the textile during drying. It is unclear if this extended viability affects biofilms in the textiles in the long term.

The described phenomena should be extendable to textile materials other than cotton and polyester. The man-made TENCEL textile is made of hydrophilic cellulose-based fibers (45). Thus, Tencel textiles supported relatively low bacterial growth when inoculated with harvested sweat, comparable to cotton (40). Nylon is also a synthetic fiber material and has a hydrophobicity between that of cotton and polyester (46), with bacterial growth showing a corresponding pattern (40). However, no significant difference in odor intensities has been observed between nylon and polyester (47). A textile material that appears to defy our hypothesis is wool, which is both hydrophobic and produces little malodor (43). However, this is due to the heterogenous nature of the wool fibers. Even though the fiber surface is hydrophobic (48), wool fibers are still very hygroscopic due to the cellulosic interior (49). This allows the wool to better retain and sequester the produced odor compounds (43), even though the fiber surface likely adsorbs more bacteria and sebum. More textile materials with different properties need to be studied to evaluate whether the observed phenomena are a general rule or apply only to cotton and polyester.

One essential property of sportswear is low hygroscopicity, which is the primary reason that polyester textiles are used for this application. However, this study indicates that the increased hydrophobicity leads directly to higher sebum absorption and therefore a higher bacterial malodor. Increasing hydrophilicity would mitigate malodor production but compromise the low hygroscopicity, which is the central feature of polyester sportswear. However, a compromise between these two properties may not be necessary. An ideal solution would be to employ fibers or fiber coatings with low surface energy, like silicones and fluorocarbons, which are both hydrophobic and lipophobic (50, 51). Such textiles have already been designed that exhibit good lipophobic and hydrophobic properties (52, 53). It is expected that such textiles would also produce less bacterial malodor during sweating due to lower sebum absorption.

### Conclusion.

Studying the differences in bacterial behavior between cotton and polyester textiles through our designed model system has given key insights into the mechanisms that influence the bacterial life cycle in clothes. The more hydrophobic textile adhered more bacteria, due to the lower energy barrier to adhesion. Stronger bacterial adhesion drives faster establishment of surface-associated biofilms. However, once the textile dries, the bacteria adhere irreversibly to the fibers, due to capillary interactions. Although the more hydrophilic cotton has a higher moisture regain, insufficient water was retained to prevent irreversible adhesion in either of the textiles. As most textiles dry before washing, preventing bacterial adhesion to the textile fibers is likely not the most effective strategy to prevent malodor production.

The more hydrophobic polyester also absorbed more sebum into the textile. The absorbed sebum provides the bacteria with a nutrient source, which causes malodor production through incomplete degradation. The higher sebum content in polyester is hypothesized to be the cause of the increased bacterial activity compared to that of cotton, indicating that this is the reason for the higher malodor production in polyester sportswear. Increasing textile hydrophilicity would likely mitigate odor production but compromise low hygroscopicity, the central feature of polyester sportswear. An ideal solution may be found creating textiles of low-surface-energy materials that are both hydrophobic and lipophobic.

The insight obtained by studying the bacterial life cycle in textiles may also be applied to other settings where bacterial colonization is an issue, especially surfaces that are periodically wetted and dried. When bacteria adhesion should be minimized, surface hydrophilicity can be optimized. Meanwhile, when bacterial viability should be minimized, the surface can be made hydrophobic to minimize water binding.

## MATERIALS AND METHODS

### Synthesis of artificial sweat and sebum.

All sweat components were bought from Sigma-Aldrich. To prepare artificial sweat, 1 liter of Milli-Q water was preheated to 37°C. The individual components were added to the water in the indicated concentrations (Table 1). The dissolved riboflavin concentration that could be achieved was 53% of that used previously, while the nicotinic acid concentration was 100-fold lower (5). After adding the solutes, pH was adjusted to 6 and the solution was sterile-filtered through a 0.22-μm Corning cellulose acetate filter.

Artificial sebum is composed mainly of a lipid extract of human adipose tissue. Human abdominal subcutaneous fat tissue was obtained from a liposuction of a 49-year-old male. Surgery was performed by the Department of Plastic Surgery at Aarhus University Hospital. Blood was separated from the fat tissue by centrifugation and the lipids were extracted by a chloroform-methanol extraction (10, 54). Adipose tissue was diluted to mass/volume fraction of 1/20 in an 8:4:3 vol% chloroform-methanol-water mixture. The cells were homogenized and lysed by sonication. After centrifugation, the chloroform phase containing the lipids was extracted and washed in 0.9% NaCl. Squalene and cholesterol were added (Table 1) and the chloroform solvent was removed by evaporation. The artificial sweat and sebum were stored at 4°C, mixed in the annotated proportions, and vortexed at 3,400 rpm for 20 s prior to use.

The lipid composition of the artificial sebum was analyzed by liquid chromatography and mass spectrometry (LC-MS), and the fatty acid composition was investigated by gas chromatography and flame ionization detection (GC-FID) on fatty acid methyl esters (FAME).

### *In vitro* inoculation of sweaty textiles.

**FIG 8:**
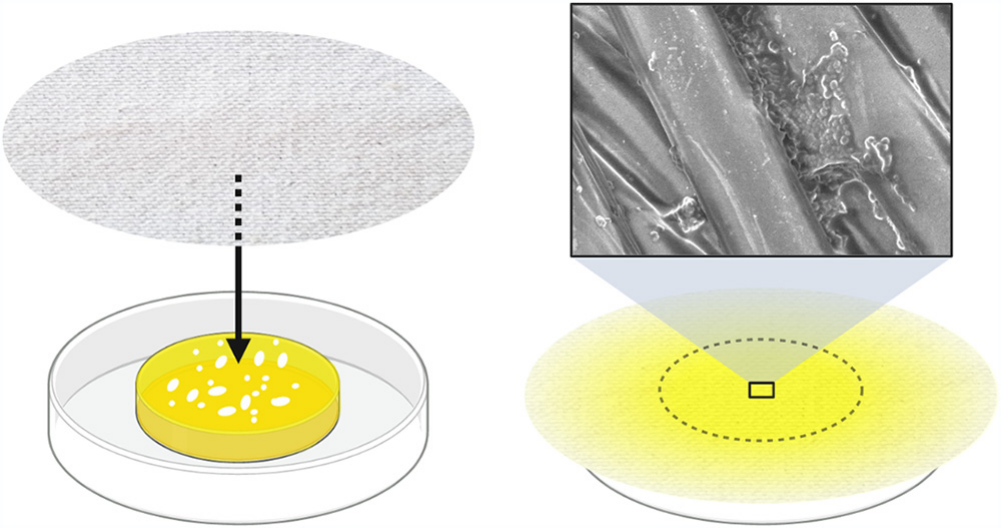
Model designed to mimic textile in contact with sweating skin. The textile is brought in contact with the artificial sweat-sebum-microbiome inoculum for 1.5 h and incubated at 37°C for 24 h.

Microorganisms were purchased from DSMZ. S. hominis DSM 20328, S. epidermidis DSM 20044, M. luteus DSM 20030, and C. jeikeium DSM 7171 were grown for 12 h at 37°C in 10 ml tryptic soy broth (TSB) plus 0.2% Tween 80. *C. acnes* DSM 1897 was grown for 84 h anaerobically at 37°C in 20 ml TSB. The bacterial species were tested individually and collectively (all together). The bacteria were washed in 0.9% NaCl and adjusted individually to an optical density (OD) of 1 in the indicated liquid. The bacteria were diluted to 2.22 × 10^8^ ml^−1^ per species in a 10-ml solution, which was placed in a 35 by 10 mm petri dish. A circular piece of pre-autoclaved textile (diameter of 11 cm) was placed on top of the petri dish (Fig. 8). The center of the textile made contact with the liquid and was inoculated for 1.5 h at 21°C. This is referred to as the inoculation step. The sweat-sebum-bacteria mixture was then removed, and the textile was subsequently incubated for 24 h at 37°C and 30% RH in an open system where the absorbed water could evaporate. This is referred to as the incubation step.

### Characterization of textile properties.

The Wilhelmy plate method was employed to investigate the fiber surface hydrophobicity of the cotton and polyester fabrics (17). A Sigma 700 tensiometer was used to measure the wetting force. Textile pieces were cut and mounted on the tensiometer, held in a rigid vertical position, and gently immersed in the liquid, with less than 1 mm below the surface. After saturation, the wetting force was recorded by the tensiometer. Wetting was first recorded in hexadecane. The textiles pieces were dried for 48 h at 37°C. The textile pieces were cooled to room temperature, and the wetting force in water was recorded. The fiber hydrophobicity was calculated from the measured wetting forces, assuming the fibers to have hydrophobicity equal to that of the macroscopic textile pieces (17).

To characterize sweat, sebum, and water uptake as well as moisture regain, artificial sweat-sebum was introduced to the textile by the inoculation and incubation step without bacteria. The textile weight was measured before inoculation, after inoculation, after incubation at 37°C and 30% RH, and after drying overnight in an oven at 105°C. To record the evaporation kinetics, the textile weight was recorded every 5 min for the first 60 min during incubation and every 10 min for the following 60 min. The textiles were weighed after 24 h of incubation, where it was assumed that equilibrium had been reached.

### Visualization of lipid distribution in textiles.

To determined how the sebum lipids distribute in the textile, lipids were stained by Nile Red and visualized by confocal laser scanning microscopy (CLSM). Artificial sweat-sebum was introduced into the textiles using the inoculation and incubation step without bacteria. A total of 100 ml 1 μg/ml Nile Red dissolved in 70 vol% ethanol was added to the textile before CLSM imaging with a Zeiss LSM 700 with a 40×/1.2 NA plan-Apochromat objective and excitation at 405 nm (for SYTO41) and 555 nm (for Nile Red). The 405-nm laser was used to visualize the textile fibers, as it induced a weak autofluorescence. Both sides of the textile were visualized, with the side initially in contact with the sweat-sebum reservoir henceforth referred to as the downfacing side and the opposing side referred to as the upfacing side.

### Bacterial adhesion to textiles.

Sweat-sebum or sweat alone was introduced to the textiles using the inoculation step without bacteria. The textiles were subsequently incubated at 37°C and for 24 h and small pieces of textile were cut from the center. The given bacterial species were cultured until they reached early stationary phase. The bacteria were pelleted and washed in 0.9% NaCl. Bacteria were resuspended in PBS and adjusted to an OD of 1. Each textile piece was submerged in 1 ml of the bacterial suspension and incubated for 1.5 h at 37°C and 150 rpm. The textiles were subsequently washed 10 times manually by pipette with 1 ml 0.05% Triton X-100 in PBS, stained with 10 μM SYTO 9, and imaged by Zeiss LSM 700 with SYTO 11/SYTO 41 settings. Nine z-stacks of 320 μm by 320 μm were taken at random locations on each side of the textiles. The bacteria in the images were counted by the Daime software (55). To compare data from individual species, the bacterial densities were normalized according to the linear normalization sum-based method (56).

### Bacterial activity in textiles.

Assessing the growth and activity of the bacteria in sweaty textiles was done by quantifying the ATP content, as done previously (57–59). Textiles were inoculated by the indicated bacterial species, or all species together, and suspended in the indicated liquid for 1.5 h. After inoculation, half of the center section of the textile (Fig. 8), which had been in contact with the inoculum, was cut out and the ATP was quantified. The other half was incubated for 24 h at 37°C, after which the ATP content was quantified. ATP was quantified using a BacTiter-Glo microbial cell viability assay from Promega. The wet textile was immersed in an Eppendorf tube with a solution of 300 μl PBS and 300 μl BacTiter-Glo reagent. Dried textiles were wetted just prior to immersion in the solution. The tubes were vortexed, the textile pieces were mixed in the solution by tweezers, and the tubes were incubated for 15 min at 21°C and 150 rpm. The tubes were subsequently vortexed again and 100 μl of the solution was added to three different wells in a 96-well plate (Nunclon Delta Surface). Luminescence intensity was recorded (VarioScan Flash, Thermo) 33 min after addition of the luciferase reagent, and the average intensity was calculated from the three wells. The recorded luminescence was converted to ATP concentration by a standard curve. The dried textiles were weighed and the ATP content was adjusted to the textile dry weight. ATP concentrations were normalized according to the linear normalization sum-based method to compare the activity of the different species.

### Bacterial adhesion to textiles before and after drying.

Textiles were inoculated with the artificial sweat-sebum and all the bacterial species together for 1.5 h. After inoculation, two pieces of textile from the center section were cut out for analysis. The rest of the textile was incubated for 24 h at 37°C, after which similarly sized pieces were cut out for analysis. Of each sample, one textile piece was washed vigorously to retain only irreversibly adhered bacteria, while the other piece was briefly wetted to allow for retention of loosely adhered bacteria. Harsh washing was done by suspending the textile piece in 1 ml 0.05% Triton X-100 in PBS and vortexing at 3,400 rpm for 30 s, before washing 10 times by pipette with 1 ml 0.05% Triton X-100 in PBS. The textiles were stained with SYBR green II RNA gel stain (Thermo Fisher Scientific) and imaged by Zeiss LSM 700 with SYTO 11/SYTO 41 settings. Nine z-stacks of 320 μm by 320 μm were taken at random locations on each side of the textiles. The biovolume of the bacteria was quantified by the Daime software.

## References

[1] Shelley WB, Hurley HJ, Nichols AC. 1953. Axillary odor: experimental study of the role of bacteria, apocrine sweat, and deodorants. Ama Arch Derm Syphilol 68:430–446. doi:10.1001/archderm.1953.01540100070012. 13091383

[2] Wang H, Newby BMZ. 2014. Applicability of the extended Derjaguin–Landau–Verwey–Overbeek theory on the adsorption of bovine serum albumin on solid surfaces. Biointerphases 9:041006. doi:10.1116/1.4904074. 25553881 PMC4286104

[3] Munk S, Münch P, Stahnke L, Adler-Nissen J, Schieberle P. 2000. Primary odorants of laundry soiled with sweat/sebum: influence of lipase on the odor profile. J Surfact Deterg 3:505–515. doi:10.1007/s11743-000-0150-z.

[4] Fredrich E, Barzantny H, Brune I, Tauch A. 2013. Daily battle against body odor: towards the activity of the axillary microbiota. Trends Microbiol 21:305–312. doi:10.1016/j.tim.2013.03.002. 23566668

[5] Harvey CJ, LeBouf RF, Stefaniak AB. 2010. Formulation and stability of a novel artificial human sweat under conditions of storage and use. Toxicol In Vitro 24:1790–1796. doi:10.1016/j.tiv.2010.06.016. 20599493

[6] Robinson S, Robinson AH. 1954. Chemical composition of sweat. Physiol Rev 34:202–220. doi:10.1152/physrev.1954.34.2.202. 13155186

[7] Stefaniak AB, Harvey CJ. 2006. Dissolution of materials in artificial skin surface film liquids. Toxicol in Vitro 20:1265–1283. doi:10.1016/j.tiv.2006.05.011. 16860531

[8] Gao B, Steenhuis TS, Zevi Y, Morales VL, Nieber JL, Richards BK, McCarthy JF, Parlange J-Y. 2008. Capillary retention of colloids in unsaturated porous media. Water Resour Res 44. doi:10.1029/2006WR005332.

[9] Mohana D, Thippeswamy S, Abhishe R. 2013. Antioxidant, antibacterial, and ultraviolet-protective properties of carotenoids isolated from Micrococcus spp. Radiat Prot Environ 36:168–174. doi:10.4103/0972-0464.142394.

[10] Callewaert C, Buysschaert B, Vossen E, Fievez V, Van de Wiele T, Boon N. 2014. Artificial sweat composition to grow and sustain a mixed human axillary microbiome. J Microbiol Methods 103:6–8. doi:10.1016/j.mimet.2014.05.005. 24858451

[11] Taylor D, Daulby A, Grimshaw S, James G, Mercer J, Vaziri S. 2003. Characterization of the microflora of the human axilla. Int J Cosmet Sci 25:137–145. doi:10.1046/j.1467-2494.2003.00181.x. 18494895

[12] James AG, Austin CJ, Cox DS, Taylor D, Calvert R. 2013. Microbiological and biochemical origins of human axillary odour. FEMS Microbiol Ecol 83:527–540. doi:10.1111/1574-6941.12054. 23278215

[13] Callewaert C, De Maeseneire E, Kerckhof F-M, Verliefde A, Van de Wiele T, Boon N. 2014. Microbial odor profile of polyester and cotton clothes after a fitness session. Appl Environ Microbiol 80:6611–6619. doi:10.1128/AEM.01422-14. 25128346 PMC4249026

[14] Wichmann S, von Koenig CW, Becker-Boost E, Finger H. 1985. Group JK corynebacteria in skin flora of healthy persons and patients. Eur J Clin Microbiol 4:502–504. doi:10.1007/BF02014433. 4065137

[15] Kwaszewska AK, Brewczyńska A, Szewczyk EM. 2006. Hydrophobicity and biofilm formation of lipophilic skin corynebacteria. Pol J Microbiol 55:189–193. 17338271

[16] Zhang Q, Li B, Sun W. 2011. Heat and sweat transport through clothing assemblies with phase changes, condensation/evaporation and absorption. Proc R Soc A 467:3469–3489. doi:10.1098/rspa.2011.0125.

[17] Hsieh Y-L, Yu B. 1992. Liquid wetting, transport, and retention properties of fibrous assemblies: Part I: water wetting properties of woven fabrics and their constituent single fibers. Textile Res J 62:677–685. doi:10.1177/004051759206201108.

[18] Gurudatt K, Nadkarni VM, Khilar KC. 2010. A study on drying of textile substrates and a new concept for the enhancement of drying rate. J Text Inst 101:635–644. doi:10.1080/00405000902732776.

[19] Wiegerink JG. 1940. The moisture relations of textile fibres at elevated temperatures. Textile Res 10:357–371. doi:10.1177/004051754001000901.

[20] Le C, Ly N, Postle R. 1995. Heat and moisture transfer in textile assemblies: part I: steaming of wool, cotton, nylon, and polyester fabric beds. Textile Res J 65:203–212. doi:10.1177/004051759506500403.

[21] Madigan M, Martinko J, Dunlap P, Clark D. 2009. Brock biology of microorganisms. Pearson Benjamin Cummings, San Francisco, CA.

[22] Bojar RA, Holland KT. 2004. Acne and *Propionibacterium acnes*. Clin Dermatol 22:375–379. doi:10.1016/j.clindermatol.2004.03.005. 15556721

[23] Marshall K, Stout R, Mitchell R. 1971. Mechanism of the initial events in the sorption of marine bacteria to surfaces. Microbiology 68:337–348.

[24] Hermansson M. 1999. The DLVO theory in microbial adhesion. Colloids and Surfaces B: Biointerfaces 14:105–119. doi:10.1016/S0927-7765(99)00029-6.

[25] Bayoudh S, Othmane A, Mora L, Ouada HB. 2009. Assessing bacterial adhesion using DLVO and XDLVO theories and the jet impingement technique. Colloids Surf B Biointerfaces 73:1–9. doi:10.1016/j.colsurfb.2009.04.030. 19493661

[26] Ripoll L, Bordes C, Marote P, Etheve S, Elaissari A, Fessi H. 2012. Electrokinetic properties of bare or nanoparticle-functionalized textile fabrics. Colloids Surf A: Physicochem Eng Asp 397:24–32. doi:10.1016/j.colsurfa.2012.01.022.

[27] Guo L, Campagne C, Perwuelz A, Leroux F. 2009. Zeta potential and surface physico-chemical properties of atmospheric air-plasma-treated polyester fabrics. Textile Res J 79:1371–1377. doi:10.1177/0040517509103950.

[28] Feng L, Li X, Song P, Du G, Chen J. 2011. Surface interactions and fouling properties of Micrococcus luteus with microfiltration membranes. Appl Biochem Biotechnol 165:1235–1244. doi:10.1007/s12010-011-9341-9. 21870124

[29] Brant JA, Childress AE. 2002. Assessing short-range membrane–colloid interactions using surface energetics. J Membr Sci 203:257–273. doi:10.1016/S0376-7388(02)00014-5.

[30] Varshney S, Sain A, Gupta D, Sharma S. 2021. Factors affecting bacterial adhesion on selected textile fibres. Indian J Microbiol 61:31–37. doi:10.1007/s12088-020-00903-5. 33505090 PMC7810813

[31] Hsieh YL, Merry J. 1986. The adherence of Staphylococcus aureus, Staphylococcus epidermidis and Escherichia coli on cotton, polyester and their blends. J Appl Bacteriol 60:535–544. doi:10.1111/j.1365-2672.1986.tb01093.x. 3528111

[32] Mitchell R, Carr CM, Parfitt M, Vickerman JC, Jones C. 2005. Surface chemical analysis of raw cotton fibres and associated materials. Cellulose 12:629–639. doi:10.1007/s10570-005-9000-9.

[33] Natsch A, Gfeller H, Gygax P, Schmid J, Acuna G. 2003. A specific bacterial aminoacylase cleaves odorant precursors secreted in the human axilla. J Biol Chem 278:5718–5727. doi:10.1074/jbc.M210142200. 12468539

[34] Bai H, Cochet N, Pauss A, Lamy E. 2017. DLVO, hydrophobic, capillary and hydrodynamic forces acting on bacteria at solid-air-water interfaces: their relative impact on bacteria deposition mechanisms in unsaturated porous media. Colloids Surf B Biointerfaces 150:41–49. doi:10.1016/j.colsurfb.2016.11.004. 27870993

[35] Torkzaban S, Bradford SA, van Genuchten MT, Walker SL. 2008. Colloid transport in unsaturated porous media: the role of water content and ionic strength on particle straining. J Contam Hydrol 96:113–127. doi:10.1016/j.jconhyd.2007.10.006. 18068262

[36] Kissa E. 1996. Wetting and wicking. Textile Res J 66:660–668. doi:10.1177/004051759606601008.

[37] Sterndorff EB, Russel J, Jakobsen J, Mortensen MS, Gori K, Herschend J, Burmølle M. 2020. The T-shirt microbiome is distinct between individuals and shaped by washing and fabric type. Environ Res 185:109449. doi:10.1016/j.envres.2020.109449. 32278157

[38] Daeschlein G, Assadian O, Arnold A, Haase H, Kramer A, Jünger M. 2010. Bacterial burden of worn therapeutic silver textiles for neurodermitis patients and evaluation of efficacy of washing. Skin Pharmacol Physiol 23:86–90. doi:10.1159/000265679. 20016250

[39] Wiksell JC, Pickett MS, Hartman PA. 1973. Survival of microorganisms in laundered polyester-cotton sheeting. Appl Microbiol 25:431–435. doi:10.1128/am.25.3.431-435.1973. 4572894 PMC380823

[40] Teufel L, Pipal A, Schuster K, Staudinger T, Redl B. 2010. Material‐dependent growth of human skin bacteria on textiles investigated using challenge tests and DNA genotyping. J Appl Microbiol 108:450–461. doi:10.1111/j.1365-2672.2009.04434.x. 19645767

[41] James A, Casey J, Hyliands D, Mycock G. 2004. Fatty acid metabolism by cutaneous bacteria and its role in axillary malodour. World J Microbiol Biotechnol 20:787–793. doi:10.1007/s11274-004-5843-8.

[42] James A, Hyliands D, Johnston H. 2004. Generation of volatile fatty acids by axillary bacteria 1. Int J Cosmet Sci 26:149–156. doi:10.1111/j.1467-2494.2004.00214.x. 18494871

[43] McQueen RH, Laing RM, Brooks HJ, Niven BE. 2007. Odor intensity in apparel fabrics and the link with bacterial populations. Textile Res J 77:449–456. doi:10.1177/0040517507074816.

[44] Liu C, Furusawa Y, Hayashi K. 2013. Development of a fluorescent imaging sensor for the detection of human body sweat odor. Sens Actuators B Chem 183:117–123. doi:10.1016/j.snb.2013.03.111.

[45] Schuster KC, Suchomel F, Männer J, Abu‐Rous M, Firgo H. 2006. Functional and comfort properties of textiles from TENCEL fibres resulting from the fibres' water‐absorbing nanostructure: a review. Macromol Symp 244:149–165. doi:10.1002/masy.200651214.

[46] Ellison A, Zisman W. 1954. Wettability studies on nylon, polyethylene terephthalate and polystyrene. J Phys Chem 58:503–506. doi:10.1021/j150516a013.

[47] Abdul-Bari MM, McQueen RH, Nguyen H, Wismer WV, de la Mata AP, Harynuk JJ. 2018. Synthetic clothing and the problem with odor: comparison of nylon and polyester fabrics. Cloth Text Res J 36:251–266. doi:10.1177/0887302X18772099.

[48] Parvinzadeh M. 2007. Effect of proteolytic enzyme on dyeing of wool with madder. Enzyme Microb Technol 40:1719–1722. doi:10.1016/j.enzmictec.2006.10.026.

[49] Li Y, Luo Z. 2000. Physical mechanisms of moisture diffusion into hygroscopic fabrics during humidity transients. J Text Inst 91:302–316. doi:10.1080/00405000008659508.

[50] Lemal DM. 2004. Perspective on fluorocarbon chemistry. J Org Chem 69:1–11. doi:10.1021/jo0302556. 14703372

[51] Zheng P, McCarthy TJ. 2010. Rediscovering silicones: molecularly smooth, low surface energy, unfilled, uv/vis-transparent, extremely cross-linked, thermally stable, hard, elastic pdms. Langmuir 26:18585–18590. doi:10.1021/la104065e. 21114260

[52] Moiz A, Padhye R, Wang X. 2018. Durable superomniphobic surface on cotton fabrics via coating of silicone rubber and fluoropolymers. Coatings 8:104. doi:10.3390/coatings8030104.

[53] Shabanian S, Khatir B, Nisar A, Golovin K. 2020. Rational design of perfluorocarbon-free oleophobic textiles. Nat Sustain 3:1059–1066. doi:10.1038/s41893-020-0591-9.

[54] Folch J, Lees M, Stanley GS. 1957. A simple method for the isolation and purification of total lipides from animal tissues. J Biol Chem 226:497–509. doi:10.1016/S0021-9258(18)64849-5. 13428781

[55] Daims H, Lücker S, Wagner M. 2006. daime, a novel image analysis program for microbial ecology and biofilm research. Environ Microbiol 8:200–213. doi:10.1111/j.1462-2920.2005.00880.x. 16423009

[56] Jahan A, Edwards KL. 2015. A state-of-the-art survey on the influence of normalization techniques in ranking: improving the materials selection process in engineering design. Mater Des 65:335–342. doi:10.1016/j.matdes.2014.09.022.

[57] Karl DM. 1980. Cellular nucleotide measurements and applications in microbial ecology. Microbiol Rev 44:739–796. doi:10.1128/mr.44.4.739-796.1980. 7010116 PMC373202

[58] Holm‐Hansen O, Booth CR. 1966. The measurement of adenosine triphosphate in the ocean and its ecological significance 1. Limnol Oceanogr 11:510–519. doi:10.4319/lo.1966.11.4.0510.

[59] Abushaban A, Mangal MN, Salinas-Rodriguez SG, Nnebuo C, Mondal S, Goueli SA, Schippers JC, Kennedy MD. 2017. Direct measurement of ATP in seawater and application of ATP to monitor bacterial growth potential in SWRO pre-treatment systems. Desalin Water Treat 99:91–101. doi:10.5004/dwt.2017.21783.

