# Supplementary material for: The Bacterial Life Cycle in Textiles is Governed by Fiber Hydrophobicity
Source: Microbiol Spectr. 2021 Oct 13;9(2):e01185-21. doi: 10.1128/Spectrum.01185-21 (PMC8515937; doi:10.1128/Spectrum.01185-21)
Supplement: SUPPLEMENTAL FILE 1 — Supplemental material. Download SPECTRUM01185-21_Supp_1_seq2.pdf, PDF file, 0.8 MB [file spectrum01185-21_supp_1_seq2.pdf]

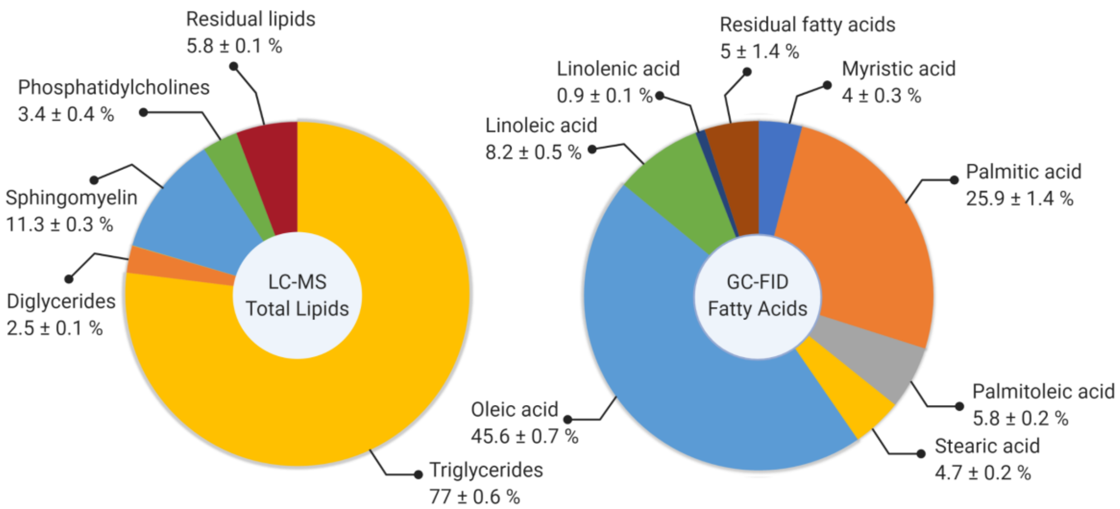


***Fig. S1. Lipid composition of artificial sebum.*** *The total lipid composition was analyzed by LC-MS, indicating that the artificial sebum was mainly compostво of triglycerides (n=3). The fatty acid composition was analyzed by GC-FID of FAME, revealing a varied composition of fatty acids (n=5).*

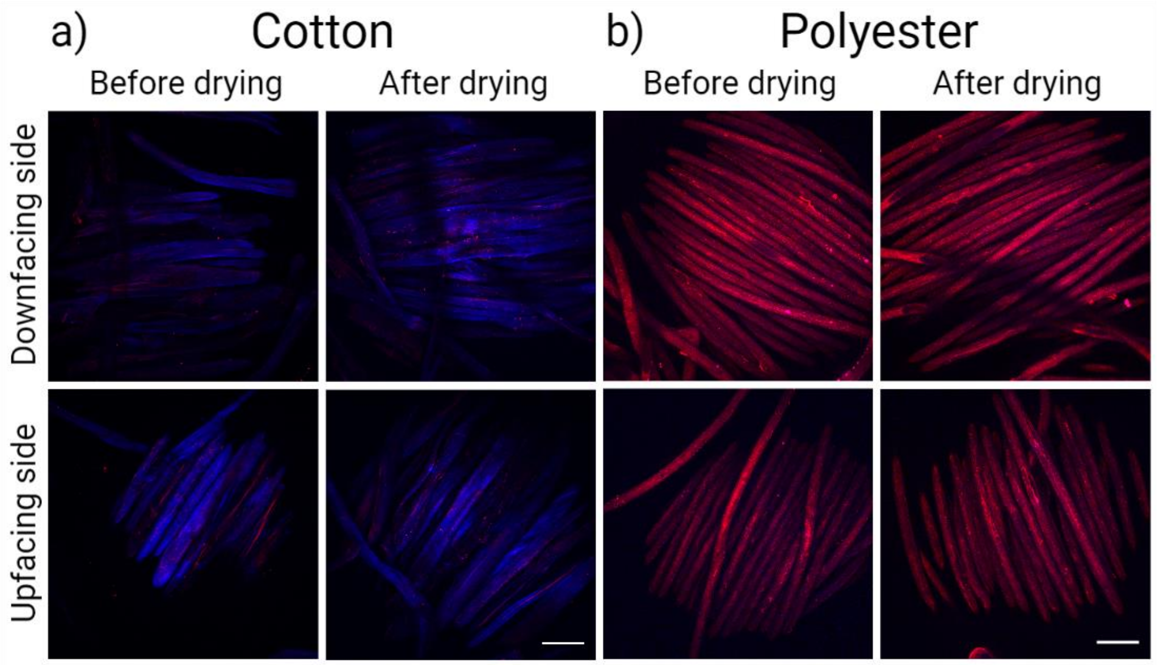


***Fig. S2. Lipid visualization in cotton (a) and polyester (b) textiles inoculated with sweat alone***. *The top row shows CLSM images of the downfacing side of the textile, before and after drying, while the bottom row shows the upfacing side. The lipids were stained by Nile Red while the fibers exhibited autofluorescence in the blue region of the spectrum.*

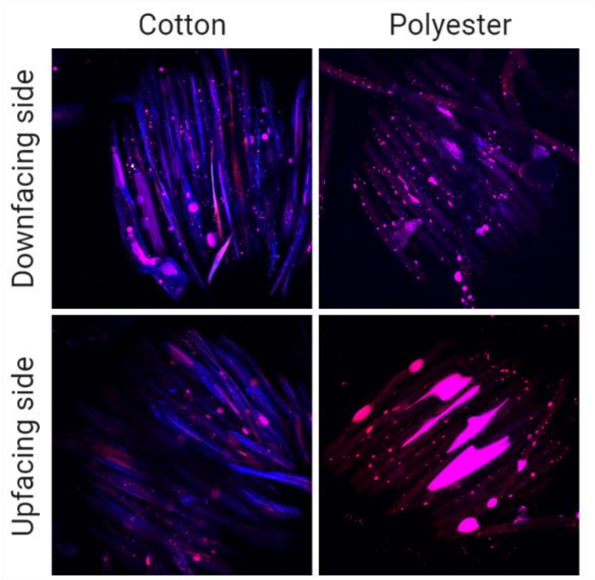


***Fig. S3. Sebum distribution in cotton (a) and polyester (b) textiles after washing with*** *Triton X-100. The top row shows CLSM images of the downfacing side of the textile while the bottom row shows the upfacing side. The sebum is stained by Nile Red while the fibers showed autofluorescence in the blue region of the spectrum*

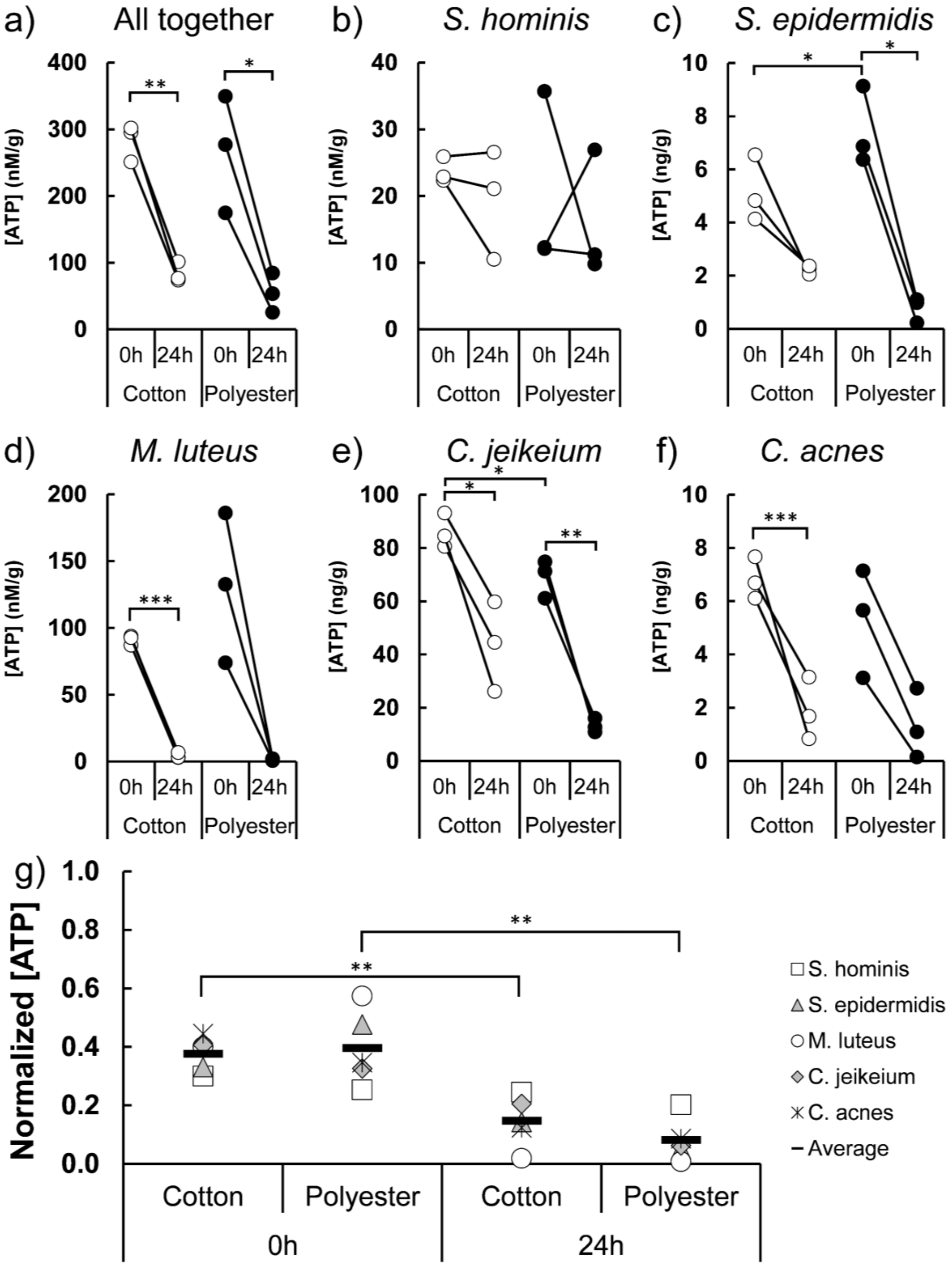


***Fig. S4. Bacterial activity in control textiles.*** *ATP concentration was measured before and after 24 hours incubation with bacteria suspended in PBS (n=3). The ATP concentration is normalized and summarized in the collective chart. Statistical significance was evaluated by a two-tailed Welch t-test*

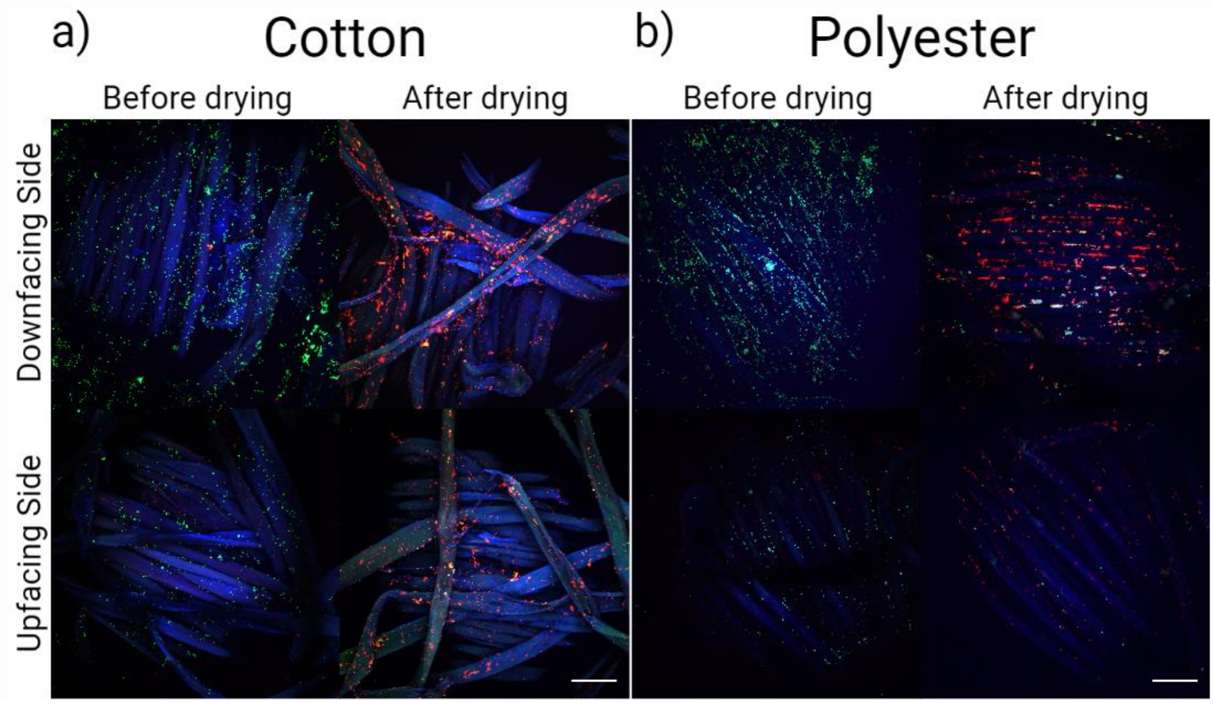


***Fig. S5. Bacterial distribution in cotton (a) and polyester (b) textiles****. The top row shows CLSM images of the downfacing side of the textile, before and after drying, while the bottom row shows the upfacing side. Bacteria were inoculated with artificial sweat-sebum and live-dead stained by SYTO 9/Propidium iodide. The textile fibers exhibited autofluorescence in the blue region of the spectrum.*

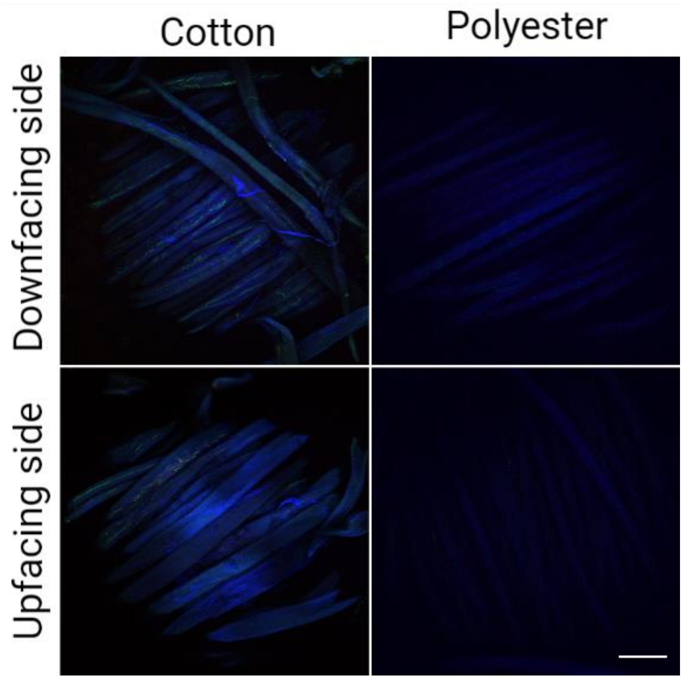


***Fig. S6. Control images of cotton and polyester textiles***. *Textiles were inoculated with artificial sweat-sebum without bacteria. The textiles were live-dead stained by SYTO 9/Propidium iodide. The textile fibers exhibited autofluorescence in the blue region of the spectrum.*
